# Targeting IRS-1/2 in Uveal Melanoma Inhibits In Vitro Cell Growth, Survival and Migration, and In Vivo Tumor Growth

**DOI:** 10.3390/cancers14246247

**Published:** 2022-12-19

**Authors:** Chandrani Chattopadhyay, Rajat Bhattacharya, Jason Roszik, Fatima S. Khan, Gabrielle A. Wells, Hugo Villanueva, Yong Qin, Rishav Bhattacharya, Sapna P. Patel, Elizabeth A. Grimm

**Affiliations:** 1 Department of Melanoma Medical Oncology, The University of Texas MD Anderson Cancer Center, Houston, TX 77030, USA; 2 Department of Genomic Medicine, The University of Texas MD Anderson Cancer Center, Houston, TX 77030, USA; 3 Department of Surgical Oncology, The University of Texas MD Anderson Cancer Center, Houston, TX 77030, USA; 4 Patient Derived Xenograft and Advanced In Vivo Models Core, and Department of Otolaryngology—Head and Neck Surgery, Baylor College of Medicine, Houston, TX 77030, USA; 5 Department of Pharmaceutical Sciences, The University of Texas at El Paso, El Paso, TX 79968, USA; 6 College of Agriculture and Life Sciences, Texas A & M at College Station, College Station, TX 77843, USA

**Keywords:** uveal melanoma (UM), liver metastasis, insulin-like growth factor (IGF-1) and its receptor (IGF-1R), insulin receptor substrates (IRS-1/2), NT157, cell growth, cell migration, cell survival, in vivo models, tumor growth

## Abstract

**Simple Summary:**

Uveal melanoma (UM) is the most common eye cancer in adults and its spreading to the liver has poor outcomes for the patients. There is currently only one therapeutic option for treating UM, hence, there is a need for a better understanding of the biology of UM metastatic spreading in order to develop novel therapies. We think that the growth factors originating from the liver contribute to UM metastasis, and here, we have investigated the role of insulin-like growth factor and its receptor (IGF-1 and IGF-1R) signaling in UM growth. We found that inhibiting the IGF-1R substrates, insulin receptor substrates-1/2 (IRS-1/2) through a small molecule inhibitor, NT157, resulted in a reduction of cell survival, migration and increased apoptosis in multiple UM cell lines. Importantly, in our in vivo models, we have shown that N157 treatment reduces UM tumor growth, indicating that targeting IGF-1/IGF-1R signaling could be a potential therapeutic strategy.

**Abstract:**

Uveal melanoma originating in the eye and metastasizing to the liver is associated with poor prognosis and has only one approved therapeutic option. We hypothesized that liver-borne growth factors may contribute to UM growth. Therefore, we investigated the role of IGF-1/IGF-1R signaling in UM. Here, we found that IRS-1, the insulin receptor substrate, is overexpressed in both UM cells and tumors. Since we previously observed that IGF-1R antibody therapy was not clinically effective in UM, we investigated the potential of NT157, a small molecule inhibitor of IRS-1/2, in blocking this pathway in UM. NT157 treatment of multiple UM cell lines resulted in reduced cell growth and migration and increased apoptosis. This treatment also significantly inhibited UM tumor growth in vivo, in the chicken egg chorioallantoic membrane (CAM) and subcutaneous mouse models, validating the in vitro effect. Mechanistically, through reverse phase protein array (RPPA), we identified significant proteomic changes in the PI3K/AKT pathway, a downstream mediator of IGF-1 signaling, with NT157 treatment. Together, these results suggest that NT157 inhibits cell growth, survival, and migration in vitro, and tumor growth in vivo via inhibiting IGF-1 signaling in UM.

## 1. Introduction

Uveal melanoma (UM) arises from melanocytes in the choroidal plexus of the eye and is biologically different from cutaneous melanoma due to different genetic alterations [1] and a strong tendency to metastasize to the liver [2,3] (observed in approximately 90% of cases with metastasis). The reason for such preferential liver seeding of UM metastatic cells is unknown. Growth factors have been shown to play a significant role in the tissue-specific homing of cancer cells [4]. Liver-borne growth factors, such as the IGF-1 may contribute to this distant metastasis to the liver and blocking these factors might have therapeutic value. IGF-1 plays a major role in tumor cell proliferation and survival, and its receptor, IGF-1R, plays a significant role in a multi-step process of cancer metastasis [5]. In UM, IGF-1R expression has shown to be independently prognostic in multivariate analysis [6]. Additionally, IGF-1 is one of the major growth factors secreted from the liver, and high IGF-1 serum levels have been shown to be associated with UM metastasis [7].

Protein expression of IGF-1R has been detected in UM tumors, and its high expression was shown to be associated with poor survival in UM patients [8,9,10]. Despite this high IGF-1R expression, few attempts have been made to target this ligand/receptor system in UM. A phase II trial that targeted IGF-1R in UM with a human monoclonal antibody from IMCLONE (NCI #8832, 2010-0451) [11] did not have any therapeutic benefit and concluded that this monoclonal antibody was not a viable treatment option. Targeting the IGF-1 pathway alone or in combination with more effective therapy regimens against metastatic UM remains to be studied [12]. IRS-1/2 are the first downstream targets of activated IGF-1R, which transmit IGF-1R mediated signals. The IRS proteins do not have their own kinase activity but act as scaffolds for signaling complexes to initiate cellular signaling pathways [13]. IRS-1/2 are intermediates of multiple receptors that control tumor progression and thus can play an important role in the response of tumor cells to different microenvironmental signals. NT157, the small molecule IRS1/2 inhibitor, developed by Dr. Alexander Levitzki, functions by serine phosphorylation and destruction of IRS-1 and IRS-2, leading to long-term blocking of IGF-1R signaling and strong inhibition of tumor growth [14] in ovarian and prostate cancers as well as BRAF inhibitor-resistant melanoma [15]. The NT compounds are highly effective in anti-cancer activity because they inhibit both the tumor growth promoted by IRS-1 and IRS-2-mediated metastasis [16]. Furthermore, inhibition of both IRS proteins precludes the compensation of one for the other [14,16].

In this study, we have utilized both the in vitro and in vivo models of UM to investigate the effect of IRS-1 inhibition on UM growth, cell survival, and migration. Our in vivo models, the CAM model and mouse subcutaneous (subQ) xenograft model [17], were previously tested and standardized for studying UM growth inhibition [18]. Here, we show that in vitro treatment with NT157 leads to inhibition of UM cell growth, induction of apoptosis, and inhibition of cell migration. More significantly, we observed inhibition of UM tumor growth on CAM as well as in our subQ mouse model with NT157 treatment.

## 2. Materials and Methods

### 2.1. Antibodies and Reagents

The IRS-1 antibody for immunohistochemistry analysis was obtained from Abcam (#ab40777; Abcam, Boston, MA, USA). Similarly, the caspase 3 antibody was obtained from Abcam (#ab184787). Cell signaling (Danvers, MA, USA) provided antibodies for caspase 9 (#9502S); hexokinase (#2867S), STAT3 (#4904S), phospho STAT 3, (#9131S), cleaved PARP (#5625S), IRS-1 for western blotting (#2382S), phospho p38 MAP Kinase (#9215S), p38 MAP Kinase (#9212S), phospho AKT (#3787S), AKT (#9272S), phospho Erk1/2(#4376S), Erk1/2 (#9102S) and IGF-1R (#3027S). Phospho IGF-1R antibody was obtained from Sigma Aldrich (SAB4300652; Millipore Sigma, Burlington, MA, USA). The Actin antibody was obtained from Santa Cruz Biotechnology (#sc-47778; Santa Cruz, Dallas, TX, USA). NT157 was obtained from Selleckchem (#NM-4126; Houston, TX, USA).

Cell migration assay plates were obtained from Corning (#354578; Corning, Glendale, AZ, USA) and the staining kit was obtained from Siemens (#B4132-1A; Siemens Health care Diagnostic Inc., Newark, DE, USA). Methylthiazole tetrazolium (MTT) reagent was obtained from EMD Millipore Corp (#475989; United Kingdom). Propidium iodide was procured from Sigma Aldrich (#81845). Ribonuclease A (RNase) was obtained from Sigma-Aldrich (#R4642).

### 2.2. Cell Culture and Treatments

Cell line Mel20–06–039, (RRID:CVCL_8473) [19,20], was obtained from Dr. Tara A. McCannel. Cell line OMM-1 (RRID:CVCL_6939) [12], Mel202 (RRID: CVCL_C301), 92–1 (RRID: CVCL_8607) [12], and Mel270 (RRID: CVCL_C302) [12] were kindly provided by Drs. Martine Jager and Bruce Ksander. UM cell lines obtained from American Type Culture Collection (ATCC, Manassas, VA, USA) were: MM28 (#CRL-3295), MP38 (#CRL-3296), MP41 (#CRL-3297), MP46 (#CRL-3298), MP65 (#CRL-3299). Additional UM cell line information is provided in the references [21,22,23,24]. Other (normal) cell lines purchased from ATCC were BJ fibroblasts (#CRL-2522), normal human epidermal keratinocytes (HaCaT;#PCS-200-011), human primary epidermal melanocytes (HEMn;#PCS-200-012).

UM cells were cultured in RPMI 1640 media with 10% FBS, glutamine, Penicillin-streptomycin, and Insulin supplement, under ambient oxygen at 37 °C. The normal cells were cultured following recommendations from ATCC.

### 2.3. Cell Line Validation

Cell lines were validated by short random repeat (STR) DNA fingerprinting techniques and mutational analysis, by the MDACC Cancer Center Support Grant (CCSG)-supported Characterized Cell Line Core, using the AmpFLSTR Identifier Kit (Applied Biosystems, Foster City, CA, USA), according to manufacturer’s instructions. The STR profiles were compared to known ATCC fingerprints (ATCC.org (accessed on 8 November 2022)), and to the Cell Line Integrated Molecular Authentication database (CLIMA) version 0.1.200808 (http://bioinformatics.istge.it/clima/ (accessed on 8 November 2022)). The STR profiles matched known DNA fingerprints or were unique.

### 2.4. Western Blotting

Cells were lysed in a buffer containing 50 mM Tris (pH 7.9), 150 mM NaCl, 1% NP40, 1 mM EDTA, 10% glycerol, 1 mM sodium vanadate, and a protease inhibitor cocktail (Roche Pharmaceuticals, Nutley, NJ, USA). Proteins were separated by SDS-PAGE with 4–20% gradient gels (Bio-Rad Laboratories, Hercules, CA, USA), transferred to a Hybond-ECL nitrocellulose membrane (GE Healthcare Biosciences, Piscataway, NJ, USA) and blocked in 5% dry milk in PBS. The membrane was then incubated with primary and secondary antibodies, and target proteins were detected with an ECL detection reagent (GE Healthcare Biosciences). For each marker, the western blots were standardized multiple times and appropriate representative blots are included in the figures.

### 2.5. Immunohistochemical Staining for IRS-1 Expression

The primary eye and liver FFPE tissues were deparaffinized and hydrated followed by blocking endogenous peroxidase activity in 3% H_2_O_2_ for 10 min. Antigen retrieval was completed with 10 mM citrate buffer in a microwave for 3 min. Samples were cooled down and washed with water followed by blocking with Biocare blocking reagent for 10 min (#BS966M, Biocare, Pacheco, CA, USA). Tissues were incubated with primary antibody at 1:100 dilution for 1 h at room temperature, followed by 5 min buffer wash. The slides were incubated with Envision plus labeled polymer, anti-rabbit-HRP (#K4011, Dako, Santa Clara, CA, USA) for 30 min at room temperature, followed by 5 min buffer wash. Next, the slides were incubated with ImmPACT VIP (#SK-4605, Vector Labs, Newark, CA, USA) (this is a purple stain) for about 3–10 min. Finally, the slides were washed, counterstained, dehydrated, and prepared for viewing.

### 2.6. Flow Cytometry Analysis (FACS) for IGF-1R Cell Surface Expression

UM cells were washed with phosphate-buffered saline (PBS) and detached by gentle pipetting. The cells were incubated with PE-conjugated mouse monoclonal anti-human IGF-1R antibody (BD Biosciences, Franklin Lakes, NJ, USA) or PE-conjugated isotype-matched control mouse antibody (IgG1k, BD Biosciences) for 1 h on ice and then washed with PBS. The cells were analyzed using FACSCalibur (BD Biosciences), and the data were analyzed using FlowJo software-FLOWJO exchange version (Tree Star, Inc., Ashland, OR, USA).

### 2.7. Cell Cycle Analysis Using Propidium Iodide Staining and FACS

UM cells were trypsinized, washed, and fixed with 70% Ethanol and kept at 4 °C overnight. Cells were centrifuged and pellets were resuspended in PBS to rehydrate for 15 min. Cells were then centrifuged at 500× *g* and treated with 200 µg/mL RNase A for 1 h at 37 °C. Cells were stained with 40 µg/mL Propidium Iodide for 20 min at room temperature. After centrifugation at 500× *g*, cells were resuspended in PBS with 0.02% EDTA for cell cycle analysis using a Beckman Coulter Galios 561 analyzer (Beckman Coulter, Brea, CA, USA).

### 2.8. Colony Formation Assay

For the colony formation assay, UM cells were seeded in 24 well plates at 500 cells/well. The next day NT157 (1 and 2.5 µM) was added to the wells. Controls were untreated cells. Cell culture media and NT157 were replaced every three days. Colonies were allowed to grow until clones in the control wells covered 70–80% of the well surface. For fixing and staining the cells, the culture media was removed, wells were washed with 1X PBS to remove dead cells and 1ml of crystal violet in 25% methanol was added. Cells were stained for 5 min at RT. Crystal violet was aspirated and the wells were washed with water until crystal violet was removed from everywhere else but the colonies.

### 2.9. Reverse Phase Protein Array (RPPA) Analysis

RPPA analyses were performed at the UT MD Anderson Cancer Center’s Functional Proteomics RPPA Core facility. Briefly, cell lysates were two-fold serially diluted for five dilutions (from undiluted to 1:16 dilution) and arrayed on nitrocellulose-coated slides. Samples were probed with antibodies using catalyzed signal amplification and visualized by 3,3′-diaminobenzidine colorimetric reaction. Slides were scanned on a flatbed scanner to produce 16-bit TIFF images of the reacting spots, and spot densities were quantified using the MicroVigene software program (Version 3.0). Relative protein levels for each sample were determined by interpolation of each dilution curve from the “standard curve” constructed by a script in R written by MD Anderson’s Department of Bioinformatics and Computational Biology. Heatmaps were generated in Cluster 3.0 (http://www.eisenlab.org/eisen/ (accessed on 8 November 2022) as a hierarchical cluster using Pearson correlation and a center metric.

### 2.10. Chicken Egg Chorioallantoic Membrane (CAM) Tumor Xenograft Model

UM cell line 92.1 engineered to express luciferase was engrafted on the CAM for seven days following previously established methods [25,26]. Briefly, embryonic day 7 eggs were inoculated with 5 × 10^5^ cells in a 1:1 Matrigel and PBS (supplemented with calcium and magnesium) solution. Eggs were randomized into two groups with 12 eggs in each set. Three days later, daily vehicle and NT157 (1 µM) treatments were topically applied. Bioluminescence imaging was performed on days 5 and 7 post-engraftment using an IVIS Lumina III in vivo imaging system (Perkin Elmer, Waltham, MA, USA). IVIS instrument exposure time was 3 s, 15 min after the addition of 15 mg/mL D-luciferin. The region of interest and total flux was determined using the Living Image Software suite. Tumors were imaged and harvested at day 7 post engraftment. Chick embryos were euthanized per AVMA guidelines. CAM tumors were fixed in 10% formalin and embedded in paraffin blocks for downstream immunohistochemical analysis. IHC was performed on paraffin sections of CAM tumors using a pan melanoma antibody cocktail (Biocare Medical, Pacheco, CA, USA).

### 2.11. Cell Viability Assays

MTT- based cell viability assays were used for estimating cell survival. UM cells were plated at a density of 1 × 10^4^ cells/well in triplicate in a 24-well plate. To assess cell viability, MTT reagent (3-(4,5-dimethylthiazol-2-yl)-2,5-diphenyltetrazolium bromide) (see above in *Antibodies and Reagents* for catalog number), dissolved in PBS, was added to a final concentration of 1 mg/mL. After 3 h the precipitate formed was dissolved in DMSO, and the color intensity was estimated in an MRX Revelation microplate absorbance reader (Dynex Technologies, Chantilly, VA, USA) at 570 nm.

### 2.12. Cell Migration Assay

Cell migration assays were performed in Boyden chambers using uncoated filters (BD Biocoat control inserts, BD Biocoat, San Jose, CA, USA). UM cells were plated in 10 cm dishes and treated overnight with NT157 (2.5 µM) or MAB391 (10 µg/mL) for 48 h. Untreated cells were used as a control. The next day, 1 × 10^5^ cells/well were plated in a serum-free medium, and the migration assay was completed with 10% FBS as a chemo-attractant and procedure as described in Chattopadhyay et al. [27]. Stained cells were photographed with a Nikon Eclipse TE2000-U microscope (Nikon Instruments Inc., Melville, NY, USA) at 20× magnification using Nikon’s NIS Elements advanced research software. To quantify migration, the cells in each filter were counted from five independent fields under the microscope at 40× magnification and the mean cell number/field was calculated. Each assay condition was tested in two replicates.

### 2.13. NT157 Treatment of SubQ UM Tumors in NSG Mice

For in vivo studies UM cell lines, 92.1 and MM28, were grown subcutaneously in NOD *scid* gamma mice. Cells of 0.5 × 10^6^ UM were suspended in 50 μL of HBSS media + growth factor reduced Matrigel (1:1 ratio) and subcutaneously injected into the right flank of each mouse. Tumors were measured by slide calipers and allowed to grow to ~75–100 mm^3^. The animals were then randomized for treatment with vehicle (40% PEG300 + 5% Tween 80 in water) or NT157 (50 mg/kg), with five animals for each treatment set. Drugs were injected intraperitoneally on alternate days for a total of three days each week until the end of the experiment. Tumor sizes were measured twice every week using slide calipers by blinded observers. All in vivo studies were performed in accordance with the accepted guidelines for housing, euthanasia, and treatment, under an Institutional Animal Care and Use Committee-approved protocol at MD Anderson.

## 3. Results

### 3.1. IGF-1R and IRS-1 Are Expressed in Uveal Melanoma

**Figure 1:**
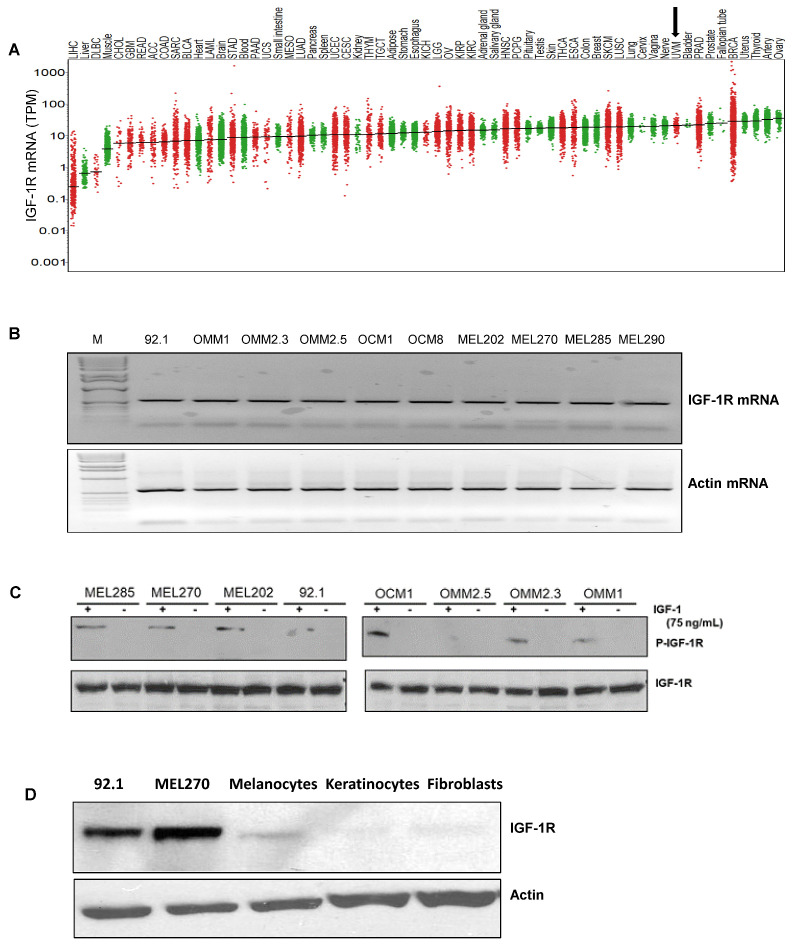

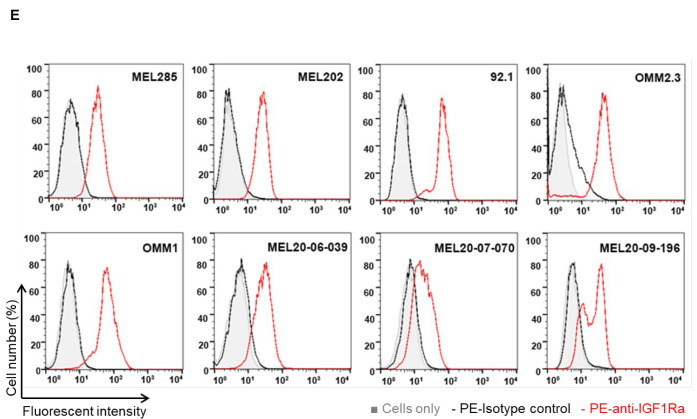
**Expression of IGF-1R in UM tumors and cell lines.** (**A**) The IGF-1R mRNA expression levels in tumor and normal tissues were compared in 33 cancer (red dots) and corresponding normal (green dots) tissues through TCGA database analysis. The expression in UM is indicated by an arrow. (**B**) RT-PCR analyses showing IGF-1R mRNA levels in 10 different UM cell lines. (**C**) Western blot analysis showing IGF-1R activation/induction in UM cell lines with 75 ng/mL of IGF-1 treatment for 10 min. (**D**) Western blot analysis for IGF-1R levels using IGF-1R antibody shows higher expression of IGF-1R protein in UM cell lines (92.1 and Mel270) vs. non-cancerous melanocytes, keratinocytes and fibroblasts. (**E**) FACS histogram plots showing cell surface expression of IGF-1R in the UM cell lines (Mel285, Mel202, 92.1, OMM2.3, OMM1, MEL20-06-039, MEL20-07-070 and MEL20-09-196) stained with IGF-1R antibody (red lines). The isotype control is represented by the black lines in the FACS histograms, and these experiments were repeated twice.

**Figure 2:**
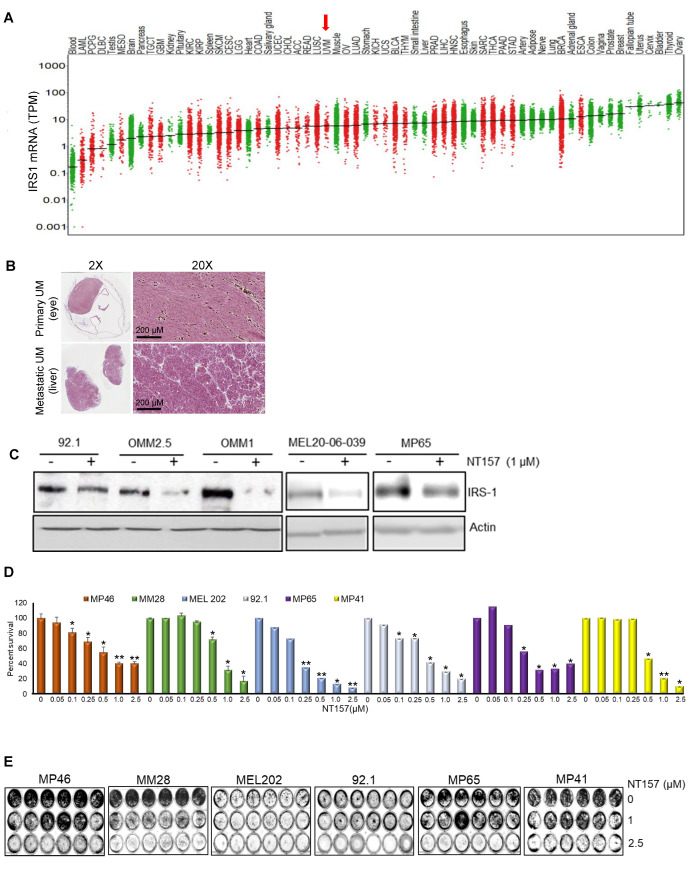

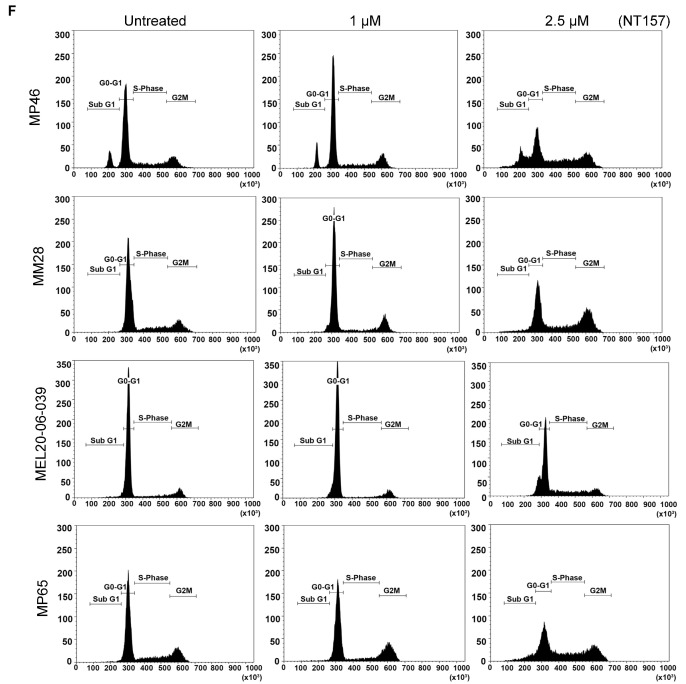

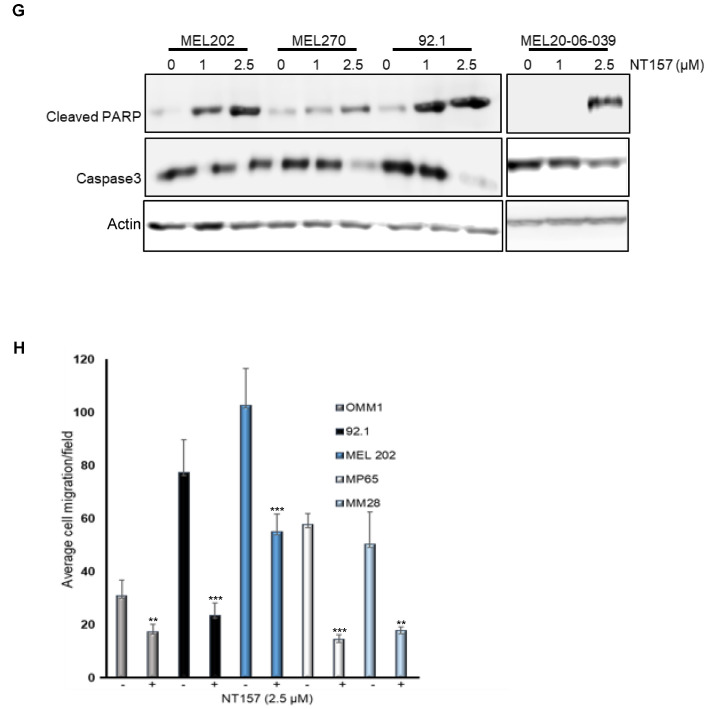
**NT157 treatment reduces IRS-1 levels leading to a reduction of cell viability, migration, and induction of apoptosis in UM cell lines.** (**A**) TCGA database analysis shows high expression of IRS-1 mRNA in UM tumors (indicated by an arrow) among 33 cancer (red dots) and corresponding normal (green dots) tissues. (**B**) Immunohistochemical staining of matched primary (eye) and metastatic (liver) UM tumor tissues using IRS-1 antibody detects IRS-1 expression (purple) in both eye and liver (both 2× and 20× magnifications are shown). (**C**) Western blot analysis of cell extracts from UM cell lines (92.1, OMM2.5, OMM1, MEL20-06-039, MP65) treated with NT157 (1 µM) and probed with anti-IRS-1 antibody shows IRS-1 protein levels decrease in UM cell lines with NT157 treatment for 48 h. (**D**) Quantification of percent cell survival using MTT-based assays in UM cell lines (MP46, MM28, MEL202, 92.1, MP65, and MP41) with (0.05, 0.1, 0.25, 0.5, 1.0, 2.5 µM) or without NT157 treatment (72 h) shows NT157 dose-dependent decrease in cell survival. The bar graphs represent a percentage of cell survival and are a mean ± SD of three independent experiments. p-values were calculated by comparing untreated controls with NT157 treatment doses (* p < 0.05; ** p < 0.01; *** p < 0.001). (**E**) Colony formation assay: Representative images of crystal violet staining shows fewer colonies formed by UM cell lines (MP46, MM28, MEL202, 92.1, MP65, and MP41) with NT157 treatment (1 and 2.5 µM for seven days) compared to untreated controls. (**F**) Representative FACS histograms of DNA content detected by propidium iodide staining to detect cell cycle status in UM cell lines (MP46, MM28 and MEL20-06-039, and MP65) show a dose-dependent increase in the number of cells in G0/G1 phase with NT157 treatment (1 and 2.5 µM for 72 h) vs. no treatment. (**G**) Western blot analysis of total cell extracts shows NT157 dose-dependent (1 and 2.5 µM for 72 h) increase in cleaved PARP and decrease in caspase 3 levels detected by respective antibodies in UM cell lines (MEL202, MEL270, 92.1, and MEL20-06-039) compared to untreated controls. (**H**) Quantification of cell migration in UM cell lines (OMM1, 92.1, MEL202, MP65, and MM28) shows a reduction in migration of cells treated with NT157 (2.5 µM) compared to untreated controls. An average of five fields of cells/filter were counted under a microscope with 40× magnification. The average number of cells counted/field were obtained in two independent experiments and the mean ± SD of these average cell counts/field were plotted as bar graphs. *p*-values were calculated by comparing untreated vs. NT157 treated cells (* p < 0.05; ** p < 0.01; *** p < 0.001).

Since IGF-1 is expressed in the highest saturation in the liver [28,29], and the liver is the primary site for UM metastasis [30], we checked the expression of the corresponding receptor IGF-1R and the immediate substrate of the receptor activation, IRS-1, in our UM cells and primary tumors. We analyzed 31 unique tumor types and their corresponding normal tissues, including UM tumors from The Cancer Genome Atlas (TCGA) data for IGF-1R gene expression. Each dot on the IGF-1R expression plot (Figure 1A) represents one tumor (red) or normal (green) sample and the samples above the median (expression line) are considered to be overexpressed compared to their corresponding normal tissues. Additionally, the IGF-1R expression levels are progressively higher moving from the left to the rightmost end of the plot. UM tumors displayed the third highest IGF-1R gene expression amongst cancer types, surpassed only by prostate adenocarcinoma (PRAD) and breast invasive carcinoma (BRCA) (Figure 1A). Next, we screened for IGF-1R RNA and protein expression in validated human UM cell lines representing known genetic backgrounds. All lines showed IGF-1R gene expression as indicated by the RT PCR analysis (Figure 1B, see full western blot images in Supplementary Materials), and phosphorylation of IGF-1R was found to be inducible by IGF-1 treatment, demonstrating it can be activated in most lines (Figure 1C, see full western blot images in Supplementary Materials). Higher expression of IGF-1R was observed in UM cells as compared to non-cancer cell lines (HEMn melanocytes, HaCaT keratinocytes, and BJ fibroblasts) (Figure 1D, see full western blot images in Supplementary Materials). The cell surface expression of IGF-1R protein on UM cells was detected by flow cytometry analysis (Figure 1E). In order to confirm the expression of downstream pathway components in UM, we investigated the expression of IRS-1, the immediate downstream effector of IGF-1R. Using TCGA data sets, we found IRS-1 transcript expression in UM human patient samples (Figure 2A). Immunohistochemical analysis of IRS-1 protein expression in a matched set of primary and metastatic UM tissues, from the eye and the liver, are shown in Figure 2B. Therefore, UM cells express both IGF-1R and IRS-1 in detectable levels.

### 3.2. IRS-1 Inhibiton Reduces Survival, Induces Apoptosis, and Inhibits Migration of UM Cells

Small molecule inhibitor NT157 inhibits its target, IRS-1, by protein degradation [14]. We treated UM cells expressing IRS-1 with NT157 to inhibit IRS-1 activity and observed a reduction in IRS-1 protein levels as shown in western blots (Figure 2C, see full western blot images in Supplementary Materials). Since the reduction in IRS-1 is expected to affect cell survival [16], we tested the effect of NT157 on UM cell survival. Through MTT assays we observed inhibition of cell survival in all cell lines tested (Figure 2D). UM cell lines varied in sensitivity to NT157 treatment (2.5 µM), ranging between 40–85% reduction in cell survival. The NT157 IC50 concentrations are 1.062 µM, 3.165 µM, 0.689 µM, 1.761 µM, 0.756 µM, and 2.234 µM for MP46, MM28, MEL202, 92.1, MP65, MP41 cell lines, respectively. We also corroborated the observation from MTT assays through the colony formation assay. We treated MEL202, 92.1, MM28, MP38, MP41, MP46, and MP65 UM cell lines with NT157 in the colony formation assay and observed inhibition of colony growth or reduction in colony number (Figure 2E). The effect of NT157 on colony formation was quantitated by counting colonies from 10 fields/sample and was plotted as a bar graph (Figure S2). We again observed varied sensitivities to NT157 among the cell lines tested.

We also observed that NT157 induces apoptosis in UM cells, as evident from the dose-dependent increase in the subG1 population upon NT157 treatment, observed through FACS analysis after propidium iodide staining (Figure 2F and Table 1). To characterize whether this G0/G1 cell accumulation is due to apoptosis, we performed molecular analysis of the apoptotic marker using western blotting. We observed increased levels of PARP processing (cleaved PARP induction) as well as caspase 3 processing (reduction in caspase 3 protein levels) after NT157 treatment of UM cell lines (Figure 2G, see full western blot images in Supplementary Materials).

As the IGF-1 pathway is a known modulator of cell migration [15], we tested the effect of IRS-1 inhibition on UM cell migration. The UM cell lines treated with NT157 overnight were subjected to a Boyden Chamber-type cell migration assay with 10% FBS as the chemo-attractant. Significant inhibition of cell migration was observed in NT157-treated UM cells (Figure 2H and Figure S1B).

We also compared the efficacy of NT157 in its ability to block different cellular/physiological processes against a neutralizing, monoclonal antibody to IGF-1R (MAB391). After confirming that MAB391 targets IGF-1R activation (Figure S1A), we compared the effect of MAB391 vs. NT157 in cell viability and migration assays. NT157 was more effective in blocking UM cell survival in an MTT-based assay in the two cell lines tested (Figure S1B) when compared with MAB391. Similarly, in an in vitro migration assay, NT157 performed better in cell migration inhibition in response to 10% FBS (Figure S1C). Overall, NT157 blocks the physiological functions of the IGF-1/IGF-1R pathway in UM cells with high efficiency.

### 3.3. Multiple Cellular Pathways Are Modulated in Response to IRS-1 Inhibition

**Figure 3:**
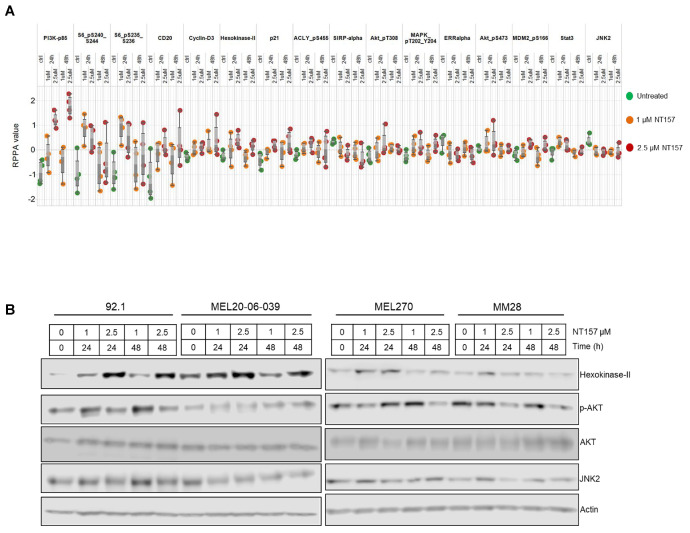
**Significant changes in cell signaling proteins detected post-NT157 treatment.** (**A**) RPPA protein profiling of four UM cell lines (MEL270, MM28, MEL-20-06-039, and 92.1; represented by each dot). The RPPA expression values are displayed on the y-axis for antibodies that show a significant difference (*p* < 0.05) compared to the control sample. Cells were treated either for 24 or 48 h with a 1 or 2.5 µM concentration of NT157. Green dots represent untreated cells; orange and red dots represent cells treated with 1 and 2.5 µM of NT157, respectively for 24 and 48 h. (**B**) Western blot analyses to validate some of the significant changes observed in RPPA using antibodies against hexokinase-II, phospho-AKT, total AKT, and JNK2 show treatment and time-dependent (1 and 2.5 µM for 24 and 48 h) upregulation of hexokinase-II and downregulation of phospho-AKT.

Since IGF-1R regulates several different signaling pathways in the cells, we wanted to understand how the major cellular signaling pathways in UM cells will be affected by NT157 treatment. Proteomic analysis with RPPA (492 antibody panel) was performed after treatment of four different UM cell lines with two NT157 doses (1 µM and 2.5 µM) and for two different time intervals (24 and 48 h). RPPA data analysis of NT157-treated vs. untreated UM cells demonstrated several changes in major cell signaling pathways. We observed dose- and time-dependent increases in protein levels of p85, the inhibitory subunit of PI3K catalytic activity compared to untreated samples. This indicates reduced activation of the PI3K pathway with NT157 treatment. We also observed a minimal increase in the activation of MAPK pathways, changes in cell cycle effectors, and an increase in hexokinase-II levels as shown in Figure 3A. The heatmap of RPPA analysis is included in Figure S3. To validate the signaling pathway changes from RPPA results, we used western blotting on an independent set of samples to detect significant changes observed. Hexokinase-II seemed to increase in an NT157 dose-dependent manner in all cell lines tested. Similarly, JNK uniformly decreased with the increase in NT157 dose. At higher concentrations (2.5 µM) and longer timepoints (48 h) AKT activation was reduced (Figure 3B, see full western blot images in Supplementary Materials). Therefore, this data shows a reduction in overall activated levels of PI3K/AKT pathway upon NT157 treatment, indicating an inhibitory effect of NT157 on UM cell survival.

### 3.4. IRS-1 Targeting Reduces UM Tumor Growth in a Chicken CAM Model

**Figure 4:**
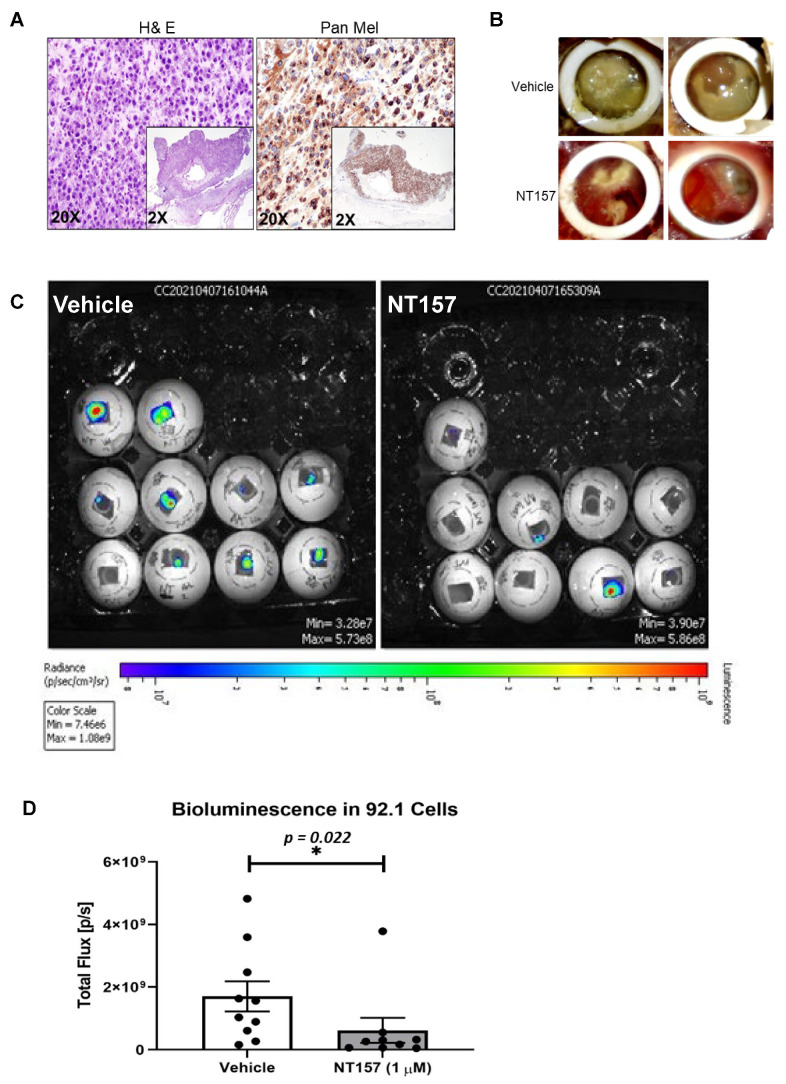
**NT157 treatment reduces UM tumor growth in the chicken CAM model.** (**A**) H&E (left) and pan melanoma cocktail (anti- HMB1, Tyrosinase, and S100 antibodies; right) staining of UM tumor tissues from 92.1 cells grown in chicken CAM model show the presence of melanoma cells. Representative (20×) and high magnification (inlay 2×) images shown as described. (**B**) Brightfield images of vehicle-treated (upper panel) and NT157-treated (1 µM; lower panel) representative tumors (two representative images per treatment) show a reduction of tumor size with NT157 treatment for four days. (**C**) Representative bioluminescence images of chicken eggs bearing luciferase-tagged UM cells (92.1) treated with vehicle controls (left panel) or NT157 (1 µM for four days) (right panel). (**D**) Tumor size of the implants was calculated as the total flux (photons per second) from the images in (**C**), which shows a reduction in tumor size with NT157 treatment; mean ± SD of three independent experiments was plotted as a graph; * p = 0.022. A student *t*-test was used for statistical analysis.

The chicken CAM has been previously used as a successful in vivo system to model UM xenograft growth [18,31]. To test the effect of IRS-1 inhibition on tumor progression in vivo, we engrafted luciferase-tagged 92.1 cells (a UM cell line) on the chicken CAM to generate UM xenografts. After confirming that the tumors generated on the CAM model are of melanoma origin by pan melanoma marker staining (HMB1, Tyrosinase, and S100) (Figure 4A), we treated the resulting tumors with different concentrations of NT157. Gross tumor analysis demonstrates a reduction in overall tumor growth in response to NT157 treatment (Figure 4B). The bioluminescence-based quantification of tumor progression showed that NT157 decreased total flux compared to the vehicle controls (Figure 4C,D). This shows a tumor size decrease with NT157 treatment. These results suggest that inhibiting IRS-1/2 can block UM tumor growth.

### 3.5. Inhibition of IRS-1 Significantly Reduces UM Tumor Growth in Mice

**Figure 5:**
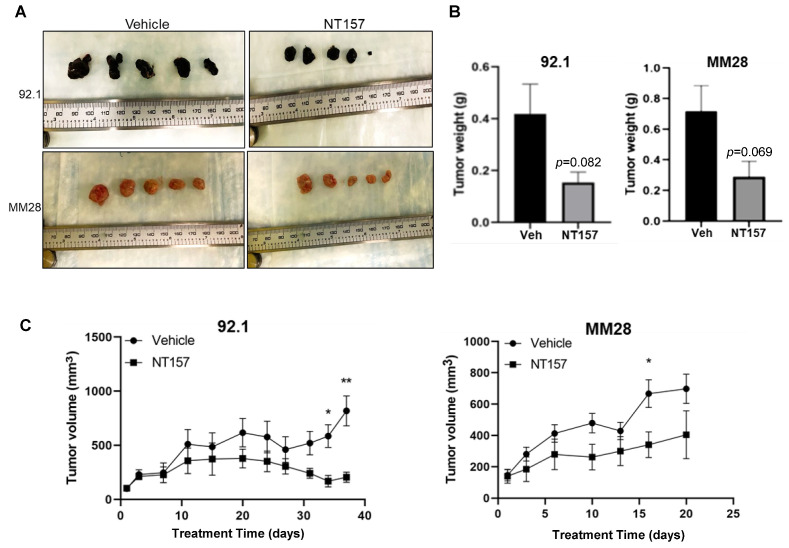
**NT157 treatment inhibits UM tumor growth in a subQ mouse model**. Subcutaneous tumors grown in NSG mice with 0.5 million UM cells 92.1 (top) and MM28 (bottom) were treated intraperitoneally with NT157 (50 mg/kg body weight), three times per week after tumors reached ~100 mm size. Tumor volume was measured twice weekly with slide calipers. When more than two tumors reached 1000 mm^3^ volume in any one group, the experiment was ended, and tumors were harvested. (**A**) Shows harvested tumors from untreated and NT157-treated groups at the endpoint. (**B**) Shows a reduction in collective tumor weight at the endpoint and (**C**) A time-dependent reduction in tumor volume with NT157 treatment (1 µM) vs. untreated controls. Student *t*-test was used for statistical analysis and the data points marked ** and * show *p* < 0.01 and <0.05, respectively.

To complement the chicken CAM model data, the efficacy of targeting IRS-1 using NT157 was also examined using subcutaneous (subQ) UM models in mice (see Materials and Methods). Xenograft tumors were generated using the UM cell lines 92.1 and MM28 in NSG mice. Five mice were treated with vehicle and five others with NT157. Treatment with NT157 strongly suppressed UM tumor growth as compared to the untreated control models (Figure 5A,C). We observed a reduction in the average UM xenograft tumor weight at the end of the experiments between the NT157 and vehicle-treated groups (Figure 5B). Therefore, in these experiments, we validated in vivo the inhibitory effect of IRS-1 blocking on tumor cell growth. There were no significant changes in the average body weights of mice in the NT157-treated vs. vehicle-treated group demonstrating that the dose of NT157 chosen for treatment was well-tolerated.

## 4. Discussion

A liver-abundant growth factor, IGF-1, has been shown to play a significant role in cell growth, apoptosis protection, differentiation, and migration [5]. Our previous attempt to target IGF-1R in UM in a phase II trial with IMC-A12, a human monoclonal antibody to the receptor [11], did not yield any responders and thus was not a viable treatment option. The success of a therapeutic strategy involving a small molecule, or an antibody depends on multiple factors like uptake, bioavailability, and stability of the agent under physiological conditions. Moreover, the efficiency of therapy also depends on compensatory and redundant pathways that are interlinked with the target of interest. Therefore, it is important to consider all these contributing factors while developing the most effective or suitable therapeutic strategy.

Here, we evaluated NT157, a small molecule inhibitor of IRS-1/2, which is the substrate immediately downstream of IGF-1R. IRS-1 inhibition successfully blocked UM cell survival, and migration and induced apoptosis in UM cells. Additionally, we observed an increase in protein levels of the p85 subunit of PI3K, a negative regulator of PI3K signaling [32]. This result indicates lower PI3K activation upon NT157 treatment. More importantly, our in vivo data from the chicken CAM and mouse subQ models show NT157 treatment-dependent tumor reduction. Since PI3K/AKT pathway promotes cell survival, reduced activation of this pathway might contribute to the observed tumor growth inhibition. We have also shown that NT157 performs better than the IGF-1R monoclonal antibody in our in vitro assays, and is, therefore, a better candidate for inhibiting IGF-1/IGF-1R signaling (Figure S1). IRS family of proteins can also be activated by some cytokines apart from IGF-1/IGF-1R [33,34]. The blocking of IRS-1/2 with NT157 probably inhibits the signal from all such feeder pathways along with IGF-1R signaling that activates the IRS proteins and may be the reason for the robust effect of NT157 observed here. Since NT157 is not a clinical compound, our study forms the basis or proof-of-concept for targeting IGF-1/IGF-1R in UM, in a clinical setting.

Previously, liver-borne growth factors, IGF-1 and HGF were proposed to be predictors for metastatic disease and potential therapeutic targets for UM [6,8,10]. Our study for the first time, clearly shows that IGF-1 not only plays a major role in the survival and migration of UM cells but also the growth of UM tumors. This is the first study to our knowledge that investigated the effect of targeting the IGF-1 pathway on UM tumor growth. Thus, our results establish IGF-1/IGF-1R signaling as an important testable therapeutic target for UM in future clinical studies.

Since Tebentafusp [35,36] is the only approved therapy for metastatic UM, and one size does not fit all, there is an urgent need to develop new therapeutic strategies. Our findings might contribute to studies in this direction. Moreover, our observation of higher IGF-1R expression in the UM cells compared to non-cancerous cells shows that targeting IGF-1R in patients may circumvent toxicity challenges. Considering the fact that single-agent therapies often do not produce durable responses in melanoma, future therapeutic strategies blocking the IGF-1/IGF-1R axis may need to consider combination therapies. Combining IGF-1R inhibition with therapies that target pathways synergistic with IGF-1R, or appropriate immunotherapies can be considered.

## 5. Conclusions

In this preclinical study, we evaluated the effect of IRS-1/2 inhibition using a small molecule inhibitor, NT157, to understand whether targeting IGF-1 signaling blocks UM growth. Our results indicate that this inhibition blocks UM cell survival and migration induces apoptosis in vitro and reduces tumor growth in vivo. Our study, therefore, supports the rationale for the development of future clinical studies based on IGF-1/IGF-1R inhibition in UM.

## Figures and Tables

**Table 1:** **Shows percent cells gated in subG1 phase with /without treatment with NT157**. The percent of cells in cell cycle phases was obtained by counting propidium iodide-stained cells after 48 h of NT157 treatment. At the higher NT157 concentration we see an accumulation of subG1 cells compared to the untreated controls.

		NT157 Treatment			
		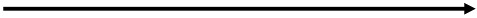			
**Percent Gated Cells in subG1 Phase**	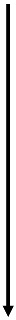	**NT157 (µM)**	**0**	**1**	**2.5**
		MP46	8.37	8.26	13.86
		MP65	1.53	1.88	7.64
		MM28	0.79	1.56	3.64
		MEL20-06-039	4.32	5.71	7.25

## Data Availability

All relevant data are available from the corresponding author upon request.

## Supplementary Materials

The following supporting information can be downloaded at: https://www.mdpi.com/article/10.3390/cancers14246247/s1, Figure S1: NT157 is more efficient in inhibiting UM cell survival and migration compared to the anti-IGF-1R antibody; Figure S2: Effect of NT157 on UM cell survival in a colony formation assay; Figure S3: Original gel and western blot images.
