# Supplementary material for: Targeting IRS-1/2 in Uveal Melanoma Inhibits In Vitro Cell Growth, Survival and Migration, and In Vivo Tumor Growth
Source: Cancers (Basel). 2022 Dec 19;14(24):6247. doi: 10.3390/cancers14246247 (PMC9777326; doi:10.3390/cancers14246247)

Supplemental Figure S1

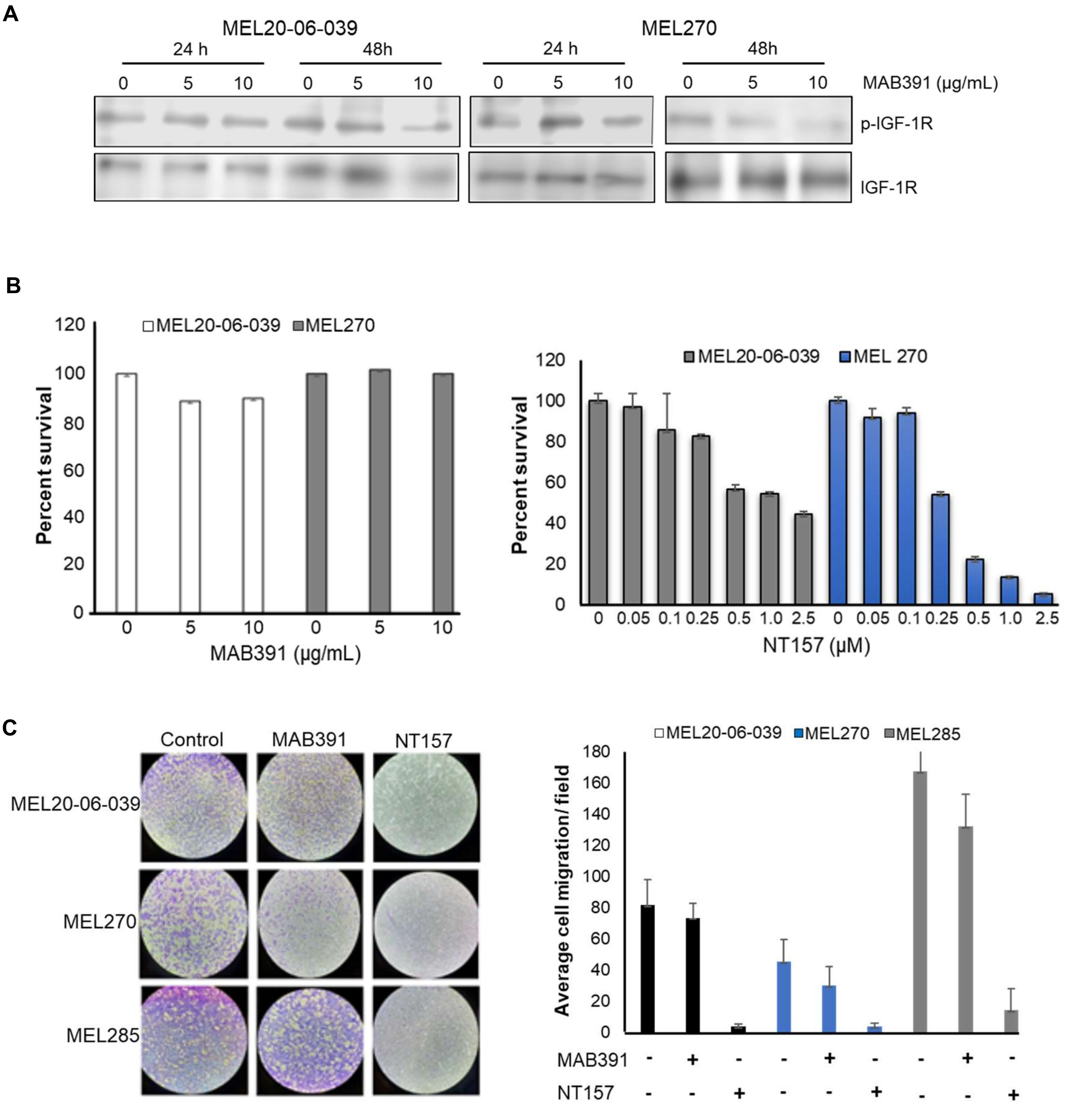

Supplemental Figure S2

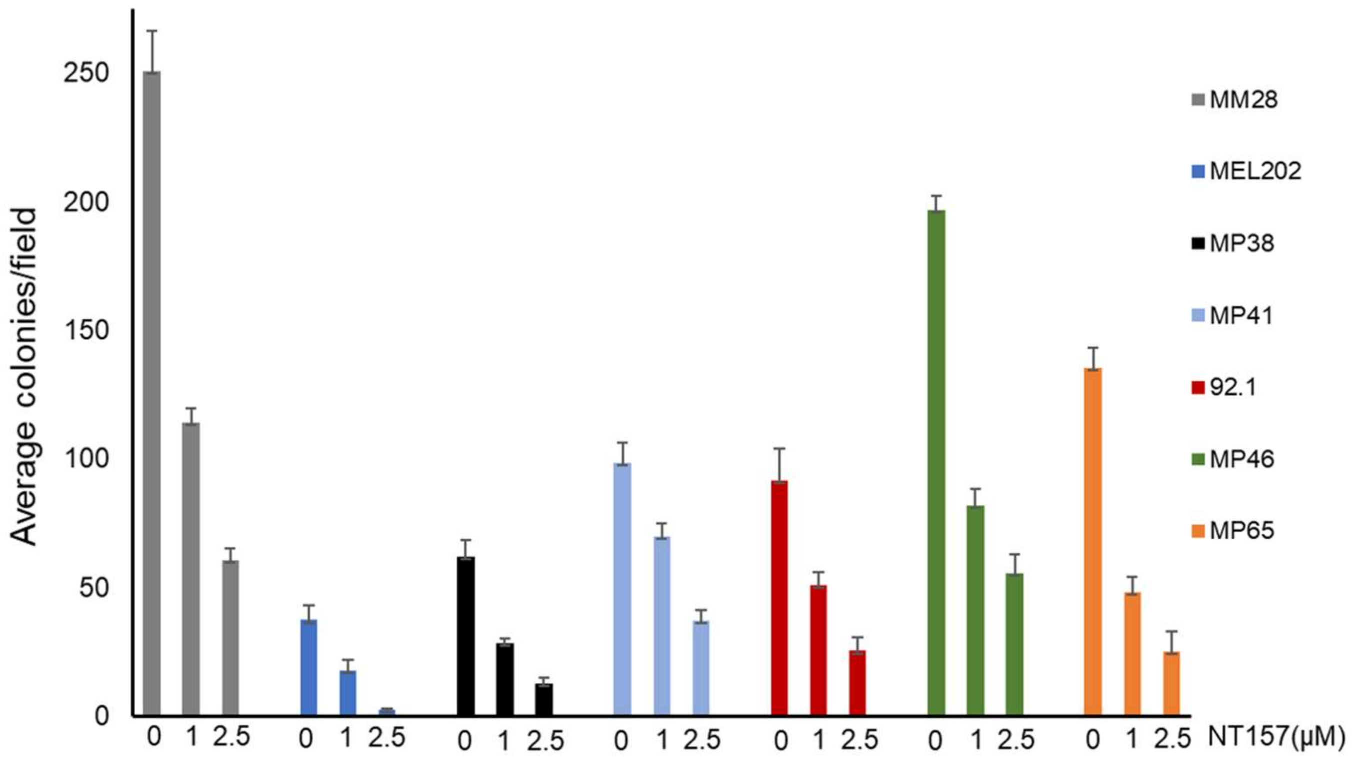

Supplemental Figure 3S: Heatmap of RPPA data set

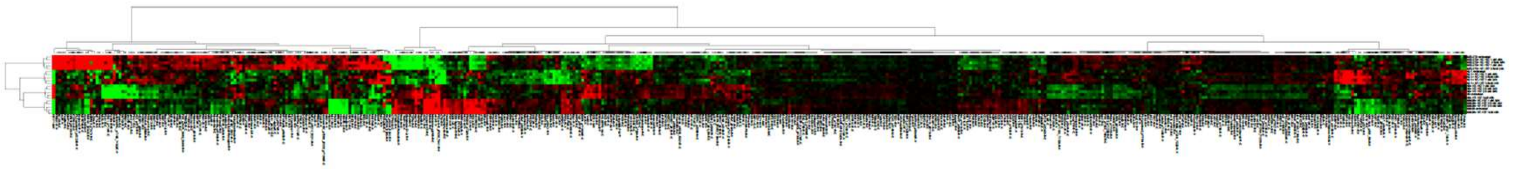

Supplemental Figure S4: Original full gel pictures

Figure 1B

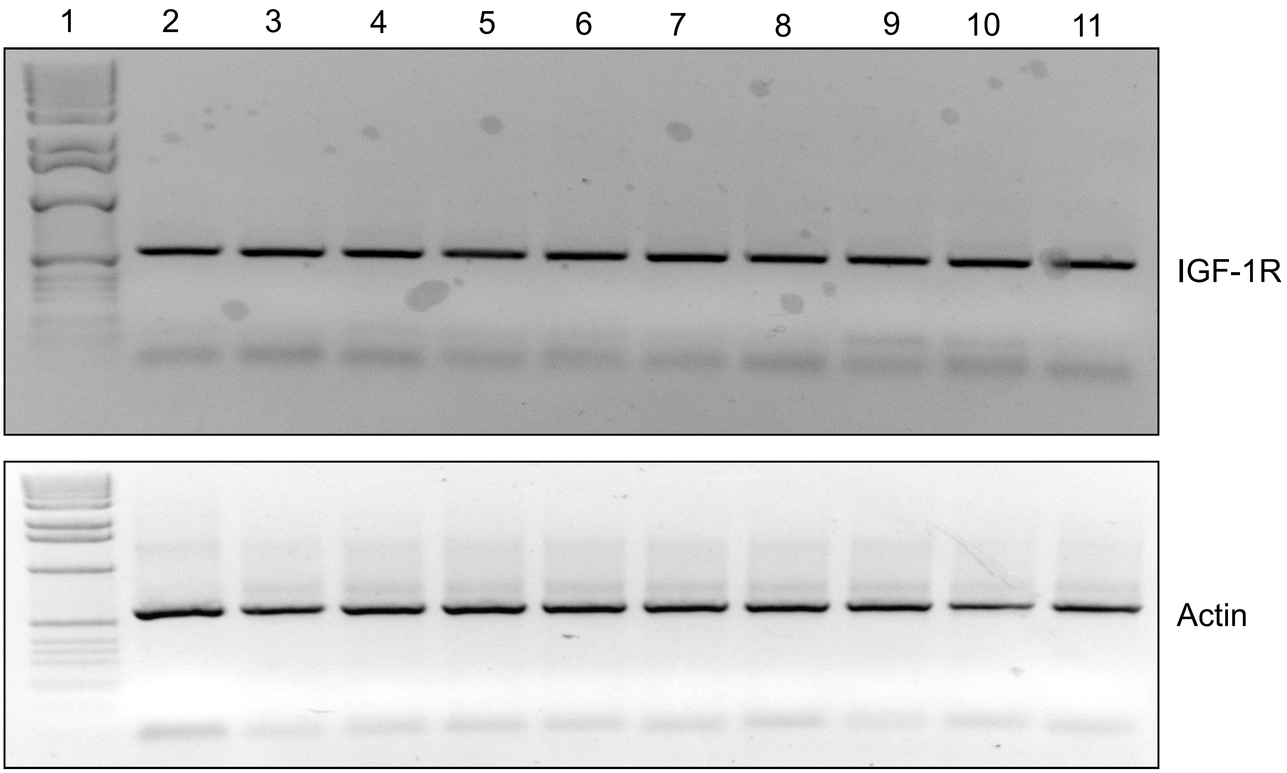

Lane	Samples
1	Marker
2	92.1
3	OMM1
4	OMM2.3
5	OMM2.5
6	OCM1
7	OCM8
8	MEL202
9	MEL270
10	MEL285
11	MEL290

Supplemental Figure S4: Original western blots

Figure 1C

Phospho IGF-1R

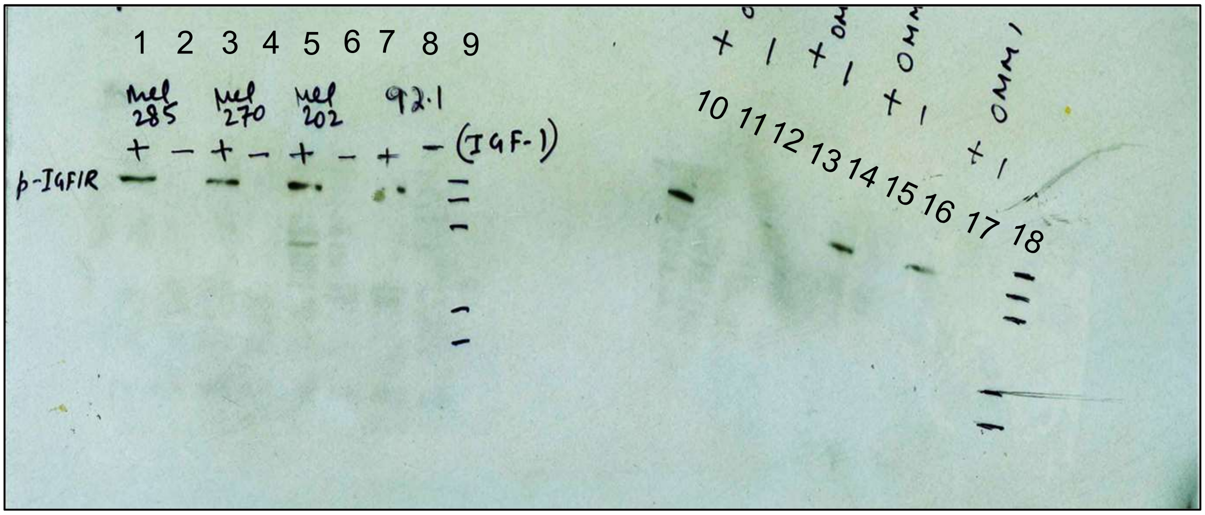

IGF-1R

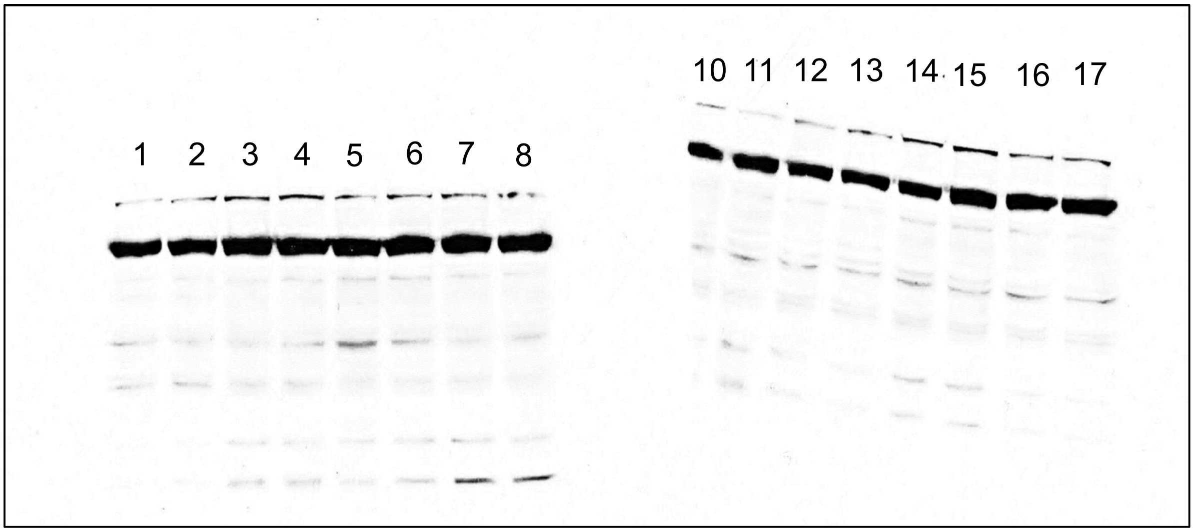

Lane	Samples
1	MEL285; 75 ng/ml IGF-1
2	MEL285; Untreated
3	MEL270; 75 ng/ml IGF-1
4	MEL270; Untreated
5	MEL202; 75 ng/ml IGF-1
6	MEL202; Untreated
7	92.1; 75 ng/ml IGF-1
8	92.1; Untreated
9	Marker

Lane	Samples
10	OCM1; 75 ng/ml IGF-1
11	OCM1; Untreated
12	OMM2.5; 75 ng/ml IGF-1
13	OMM2.5; Untreated
14	OMM2.3; 75 ng/ml IGF-1
15	OMM2.3; Untreated
16	OMM1; 75 ng/ml IGF-1
17	OMM1; Untreated
18	Marker

Supplemental Figure S4: Original western blots (continued)

Figure 1D

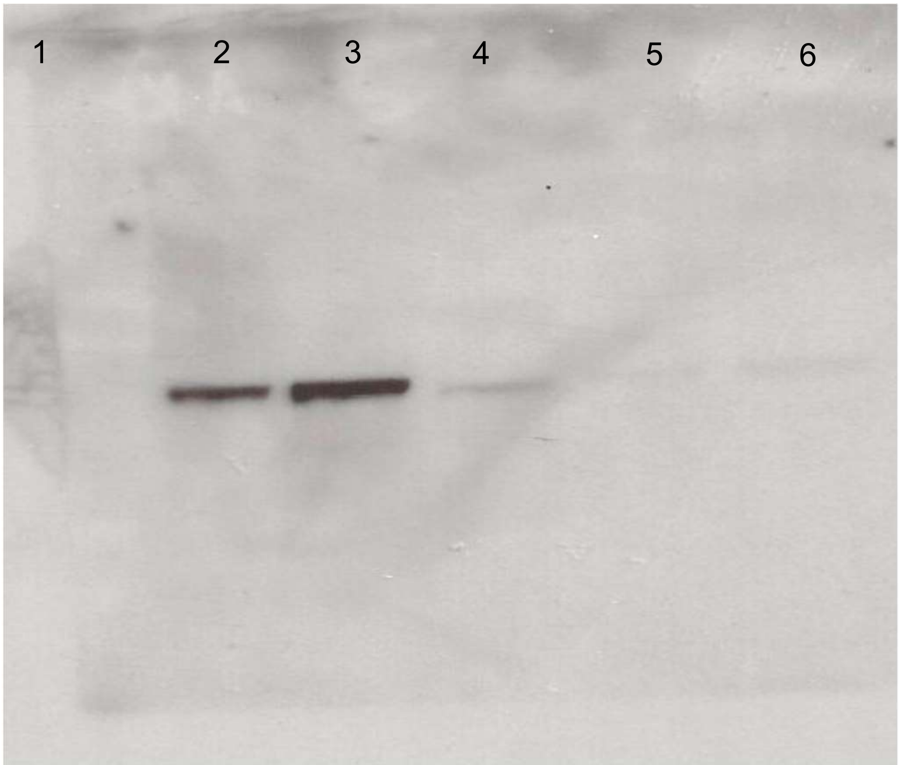

IGF-1R

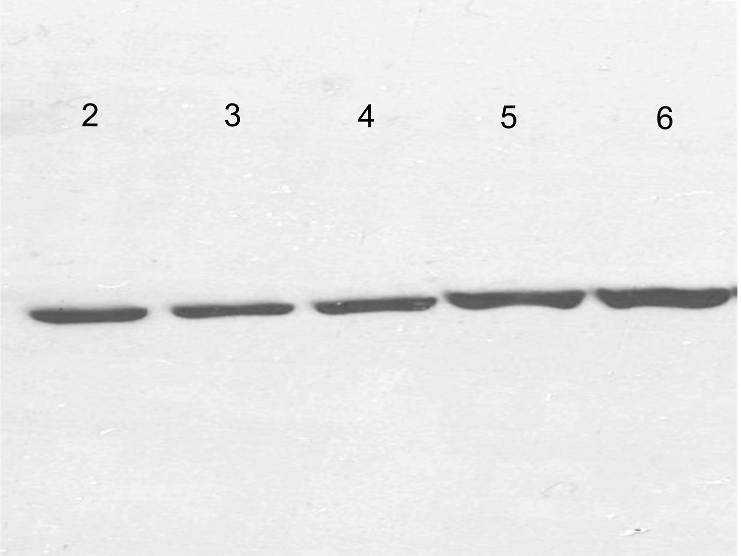

Actin

Lane	Samples
1	Marker
2	92.1
3	MEL270
4	Melanocytes
5	Keratinocytes
6	Fibroblasts

Supplemental Figure S4: Original western blots (continued)

Figure 2G

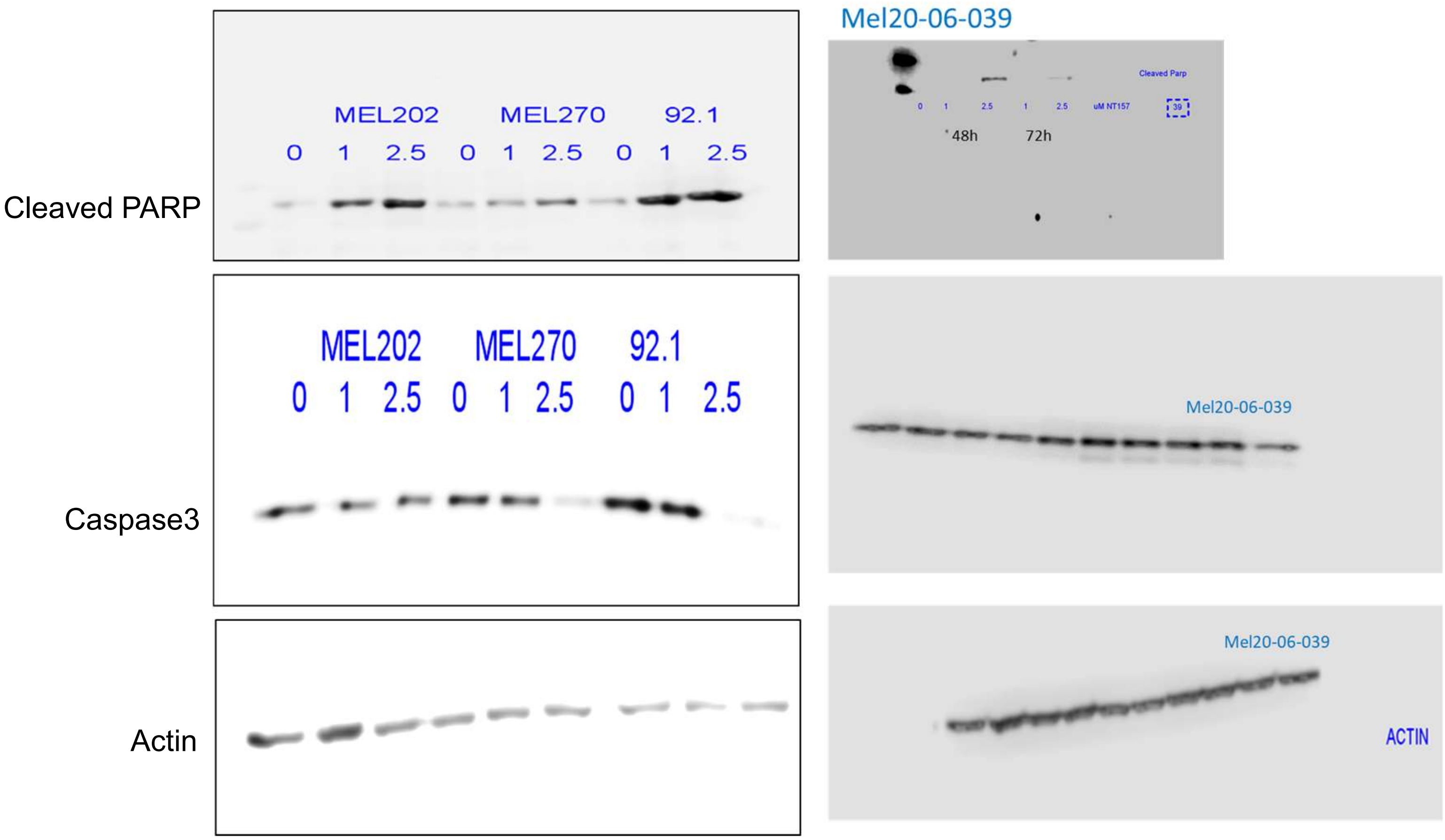

Supplemental Figure S4: Original western blots (continued)

Figure 3B

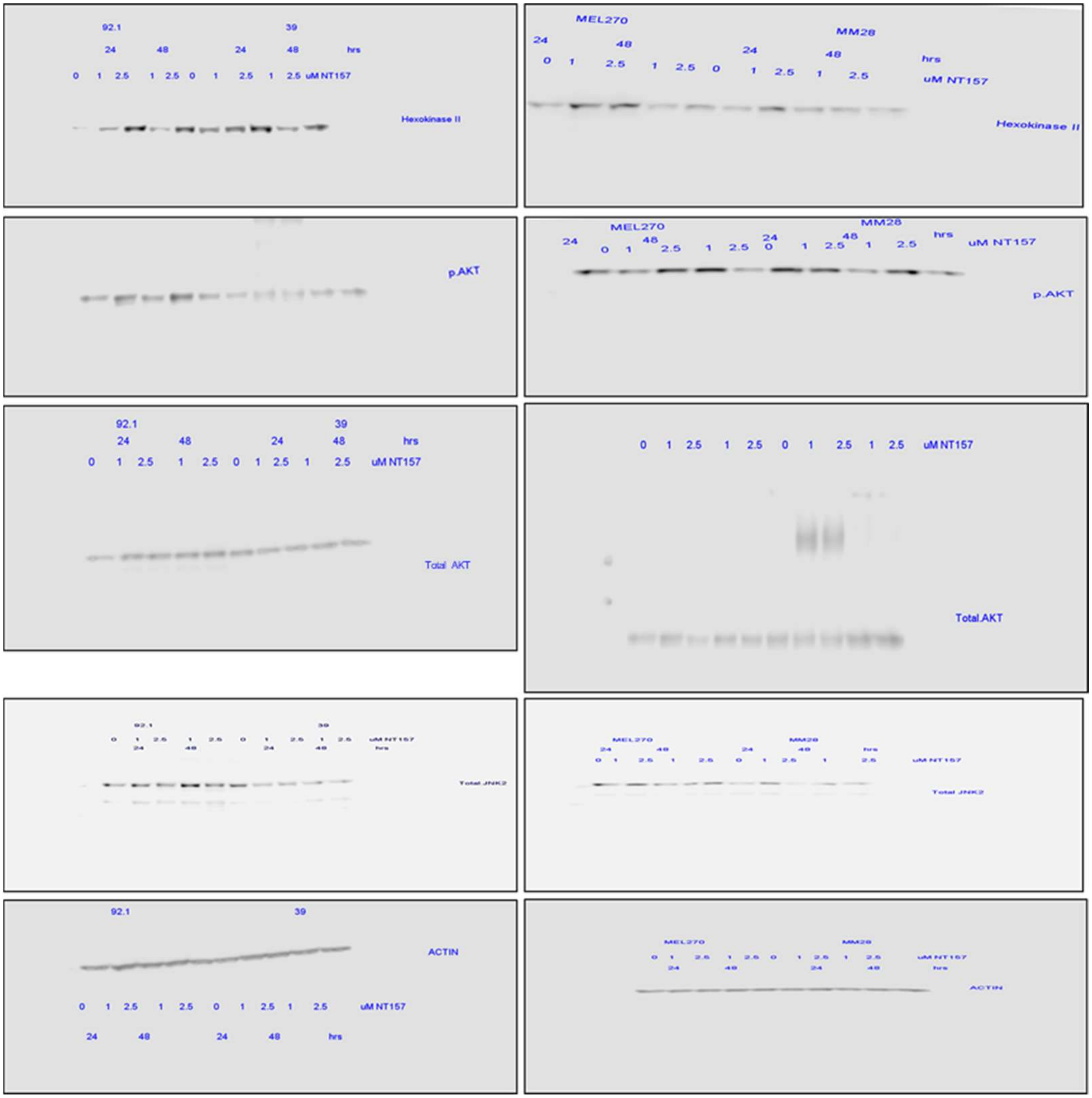

Supplement: Supplementary file 1 [file cancers-14-06247-s001.zip › cancers-2075315-supplementary.pdf]
